# Fecundity and Nutrient Deficiency Following Obesity Treatment: Implications for Young-Onset Cancer Risk in Offspring

**DOI:** 10.3390/cancers16173099

**Published:** 2024-09-06

**Authors:** Savio George Barreto, Chris Moy, Stephen J. Pandol, Lilian Kow

**Affiliations:** 1 College of Medicine and Public Health, Flinders University, Adelaide, SA 5042, Australia; lilian.kow@flinders.edu.au; 2 Flinders Medical Center, Division of Surgery and Perioperative Medicine, Flinders University, Adelaide, SA 5042, Australia; 3 Arkaba Medical Centre, Adelaide, SA 5063, Australia; chrismoy@optusnet.com.au; 4 Cedars-Sinai Medical Center, Division of Digestive and Liver Diseases, Los Angeles, CA 90048, USA; stephen.pandol@cshs.org; 5 Adelaide Bariatric Centre, Flinders Private Hospital, Bedford Park, Adelaide, SA 5042, Australia

Young-onset adult cancers have been an emerging problem over the last three decades in Australia [1,2], as well as globally [3]. Their aetiologies have remained unclarified. Common cancer-associated environmental factors (such as smoking, obesity and alcohol consumption) have been proposed to play a causative role in these cancers [4]. However, with a steady, or even declining, rate of similar cancers in people above the age of 50 years old, the mechanism of selective virulence of these environmental factors that contributes to young-onset carcinogenesis needs to be determined. Building on our understanding of chronic disease [5] and cancer timelines [6,7], the Perinatal and Early Life Influences on Cancer (PELICan) hypothesis was postulated [8,9] to help reconcile the variable effects of the common triggers (for carcinogenesis) on individuals. 

The Great Famine in China (1959-61) remains a significant event in human history. While there are many economic lessons to learn from this man-made catastrophe [10], we sought to explore the impact of perinatal malnutrition in its most extreme form on the risk of developing young-onset cancers. We interviewed survivors of the famine identified by the China Health and Nutrition Survey (CHNS). A significant increase in the risk of development of young-onset cancer was noted amongst individuals whose perinatal period encompassed the first 3 years after the famine, i.e., 1962–1964 (RR 2.08; 95% CI 1.04, 4.34; *p* = 0.043). Additionally, these individuals, as well as those whose perinatal period encompassed the ensuing 3-year period (i.e., 1965–1967), were noted to have a significantly increased risk of young-onset genitourinary cancers [(RR 13.8; 95%CI 2.68, 253; *p* = 0.012) and (RR 12.3; 95%CI 2.16, 231; *p* = 0.020), respectively] [11]. This finding poses the following question: How are findings from a famine that occurred nearly 70 years ago relevant to us today? 

Australia had the ninth highest proportion of overweight or obese people aged ≥ 15 years old amongst the twenty-one Organisation for Economic Co-operation and Development (OECD) member countries in 2021 [12]. As per the Australian Institute of Health and Welfare (AIHW), 67% of adult Australians are overweight or obese [12]. Bariatric and metabolic surgery have repeatedly been proven to result in numerous health benefits, ranging from weight loss and a reduction in musculoskeletal complaints to better glycaemic control and the need for fewer medications in type II diabetics [13], as well as to a reduction in cancer risk [14]. The findings of the recent BAMBINI multicentre, open-label randomised controlled trial [15] have confirmed the success of bariatric surgery in establishing spontaneous ovulation in women with polycystic ovarian syndrome (PCOS), obesity, and oligomenorrhoea or amenorrhoea. Previous studies had already documented spontaneous conception in females who were previously unable to conceive [16].

The success of gastric bypass as an option for weight loss surgery is measured by the acute loss of weight accompanied by macro- and micronutrient deficiency resulting from decreased oral intake and/or malabsorption [17]. Obese patients may be malnourished to begin with, often experiencing essential micronutrient deficiencies [18]. Bariatric and metabolic surgery can, therefore, further affect the microelement metabolism, predisposing these individuals to an increased risk of nutritional deficiencies [18]. The macro- and micronutrient deficiencies following bariatric and metabolic surgery persist even after 1 year, irrespective of whether the patient underwent a malabsorptive (e.g., gastric bypass) or a restrictive procedure (e.g., sleeve gastrectomy) [19]. While it may appear preposterous to compare outcomes during a famine with those following bariatric surgery, it is important to understand that these are not entirely disparate situations [20,21]. The evidence from the study by Shuai et al. [11] identified a steadily increasing risk in the period that immediately followed the famine—a time when access to nutrition started to improve. 

Many key organisations have recognised the significance of the increased propensity for conception, as well as the nutrient deficiencies that are especially significant in the first-year post bariatric surgery, and recommended that conception be delayed until at least 12 to 18 months post surgery, or until weight stabilisation occurs [22,23]. The effects of pregnancy after bariatric surgery on the child are numerous, ranging from being small for their gestational age (SGA) [24] to obesity and adverse psychomotor and behavioural outcomes in childhood [25]. In Australia, bariatric surgery is predominantly performed in the private sector [26]. Some patients may choose to terminate their private health insurance coverage after the operation, and not all patients are committed to receiving long-term follow-up, even though it is recommended following bariatric and metabolic surgery, from their specialist or multidisciplinary team. This phenomenon of loss to follow-up post bariatric surgery is a global problem [27,28]. The lack of follow-up results in an inability to reinforce the avoidance of conception in the period of catabolism and nutritional deficiency that accompanies weight loss in the first year post bariatric surgery. It also leads to an inability to provide important advice on the maintenance of optimal nutrient intake. In fact, even in patients who do undergo follow-up post surgery, there are issues involved in assessing change in dietary intake and appetite [29]. Similarly, the effect of glucagon-like peptide-1 (GLP-1) receptor agonist-induced loss of weight and consequent improvement in fertility [30], as well as the likelihood of conception’s relationship with the risk of young-onset cancer in offspring, has not been studied.

A prospective study examining the effects of macro- and micronutrient deficiency in mothers post bariatric surgery or the glucagon-like peptide-1 (GLP-1) receptor agonist and its effects on the risk of young-onset carcinogenesis in offspring conceived in the first year post surgery would only yield results decades from now. Hence, existing knowledge and insights need to inform current practices and patient education. There is a need to reinforce the published guidelines [22,23]. Females need to be informed through public health strategies. Appreciating that the only link to the healthcare system for a lot of patients will be their general practitioners (GPs), there is a need to provide GPs with both this information and the resources and support required to enforce best practices post bariatric and metabolic surgery.
